# Hollow carbon fiber wrapped by regular rGO wave-like folds for efficient solar driven interfacial water steam generation

**DOI:** 10.1038/s41598-024-64144-y

**Published:** 2024-06-18

**Authors:** Jie Yang, Peiqi Liu, Zhiyuan Fan, Yingying Li, Hongtao Qiao, Xingyu Xu, Sheng Han, Xidong Suo

**Affiliations:** 1 Department of Chemistry, Xinzhou Normal University, Xinzhou, 034000 Shan Xi China; 2 Leicester International Institute, Dalian University of Technology, Panjin, 124221 Liaoning China; 3 School of Chemical and Environmental Engineering, Shanghai Institute of Technology, Shanghai, 201418 China

**Keywords:** Solar driven interfacial evaporation, Wavy-like fold interface, Graphene oxide, Hollow carbon fiber, Seawater desalination, Materials for devices, Energy harvesting, Renewable energy

## Abstract

Efficient seawater desalination is an effective way to solve the shortages of fresh water and energy but with limitations of the low fresh water production rate and high cost. Here, a hollow carbon fiber (HCF) wrapped by regular reduced graphene oxide (rGO) wave-like folds (rGO@HCF) is prepared on account of the differences in thermal shrinkage performance between graphene oxide (GO) and willow catkins fiber. Under one sun irradiation (1 kW m^−2^), the dry and wet surface temperature of the resulting evaporator reached up to 119.1 °C and 61.7 °C, respectively, and the water steam production rate reached 3.42 kg m^−2^ h^−1^. Also, for the outdoor experiment, the rGO@HCF exhibits good evaporator performance which reach up 27.8 kg m^−2^ day^−1^. Additionally, rGO@HCF also shows good seawater desalination performance and excellent durability for longtime work. DSC results indicate that the evaporation enthalpy of bulk water and adsorbed water decreased from 2503.92 to 1020.54 J g^−1^. The excellent evaporating performance is mainly attributed to the regular wave-like microstructure surface of the HCF, which can enhance the light absorption, reduced the vaporization enthalpy of the adsorption water. The findings not only introduce a novel approach for agricultural utilization, but also establish a crucial theoretical foundation for the design of regular wave-like microstructures.

## Introduction

Water and energy are crucial for human survival. Population growth and industrialization have exacerbated the scarcity of energy and freshwater resources. Currently, over 30% of the world’s population is facing the threat of severe water scarcity^1^. Developing and utilizing clean renewable energy to produce clean water has become a strategic task for many countries around the world^2,3^. Solar energy, as a sustainable and clean energy source, has garnered significant interest to address water crises^4,5^. In 2014, Hadi Ghasemi developed an innovative interfacial evaporation system, in which solar light is absorbed by photothermal conversion materials and rapidly converted into heat energy at the water–air interface, achieving efficient evaporation of water^6^. For this system, the performance of the evaporator mainly depends on the solar light absorption and heat conversion capabilities of the photothermal conversion materials^4,7–9^. With the characteristics of wide spectral absorption (250–2500 nm)^7^, low-cost to production^10^, easily scale up to manufacture^10,11^, stable physical and chemical properties^11^ and wide range of raw material sources^12^, carbon materials have been widely investigated for utilization in the field of solar driven interfacial steam generation^4,13–15^.

Due to unique original structure, low cost and widely distributed in the world^16–19^, many biomass-based evaporators with excellent steam generation performance have been successfully developed by direct carbonization process, such as carbonized mushroom^20^, carbonized sunflower heads^21^, willow-catkins-based hollow carbon fiber^14^, carbonized wood^22^, carbonized quinoa bran based cellulose wrapped carbon fiber^23^, carbonized toilet paper-based porous hollow carbon fiber^9^. According to the reported reports^24,25^, materials with nano or microstructures can significantly improve light absorption performance and photothermal conversion efficiency. Recently, hierarchical micro-/nanostructured cuttlebone^26^, nanostructured Al–Zn alloys film^27^, GO coated silicone sponges with the nanosizing roughness skeleton^28^ exhibit excellent light adsorption ability (over 95%), and good evaporation rate. For biomass materials, direct carbonization process is most common and simple method to translate it into solar driven steam evaporator. Whereas the surface or interfacial structure of the resulting carbon materials is difficult to control for more better light adsorption, it would limit the performance of the resulting evaporator.

To address the difficult of control the surface structure, here, we firstly demonstrate a novel scheme to control the surface structure of the resulting hollow carbon fiber. Regular rGO wave-like folds pattern is successfully formed on the surface of the hollow carbon fiber based on the thermal shrinkage differences of the GO and WCF. The light adsorption of resulting evaporator is largely improved, increasing from 65.55 to 90.86%. Under one sun (1 kW m^−2^), weight loss rate of the container was reach to 3.42 kg m^−2^ h^−1^ with the surface temperature of 61.7 °C. Importantly, the prepared evaporator exhibits good outdoor evaporating and longtime work abilities. This work not only provides a new method for carbon fiber surface structure containment, but also provide a way for fiber-like agricultural utilization in the field of the solar driven interfacial steam generation.

## Experimental section

### Materials

Waste willow catkins were harvested from the Park of Xinzhou (Shanxi, China) and contaminants were removed through multiple washes. Graphene oxide (GO) aqueous solutions at a concentration of 2 mg mL^−1^ was provided from the Institute of Coal Chemistry, Chinese Academy of Sciences (Taiyuan, China). Anhydrous ethanol, glacial acetic acid, and sodium chlorite were procured from the Shanghai Macklin Biochemical Co., Ltd. (Shanghai, China). Seawater was collected from the Yellow Sea near Qingdao, Shandong, China. All reagents were utilized as procured without additional purification. A pure water system was employed to generate deionized water for use in the experiments.

### Preparation of homogeneous suspensions of composites willow catkins/GO

A homogeneous suspension of willow catkins was prepared by following methodologies established in prior studies^14,29^. Initially, 2 g of the purified willow catkins were immersed in an aqueous sodium chlorite solution (1 wt%, adjusted to pH 4.5 with glacial acetic acid) and subjected to mechanical stirring at 80 °C for 2 h. Subsequent rinsing with deionized water was performed until a neutral pH was achieved. To ensure further removal of residual impurities, the catkins underwent three successive washes with anhydrous ethanol and were subsequently dried in a blast oven at 60 °C. The dried willow catkins were then subjected to high-speed stirring (4000 rpm) for 1 h and dispersed in 200 g of anhydrous ethanol to generate a willow catkins suspension (WC suspension, as depicted in Supporting information Fig. S1). To this suspension, a measured amount of graphene oxide (GO) dispersion was introduced and stirred for 15 min, yielding a composite WC/GO suspension.

### Preparation of rGO@HCF composite membranes

Following the protocol stated in “Preparation of homogeneous suspensions of composites willow catkins/GO” section, the WC/GO suspension was subject to vacuum filtration, yielding a filter cake that was subsequently dried for 24 h at 60 °C. The resultant cake was sandwiched between alumina ceramic plates and introduced into a tube furnace under a nitrogen atmosphere. A controlled heating rate of 5 °C min^−1^ culminated in a temperature of 900 °C, at which carbonization occurred for 2 h. Upon completion, the system was allowed to return to ambient temperature, giving rise to the rGO@HCF material. Control samples, generated by direct carbonization of willow catkins devoid of GO, were denoted as HCF. Samples incorporating GO were designated rGO@HCF_0.03_ and rGO@HCF_0.06_, representing the GO mass ratio in the initial solid mixture. It is pertinent to mention that attempts to filter higher concentrations of GO suspensions resulted in impractical filtration durations, extending beyond ten days. Consequently, this study did not extend to the fabrication of membranes with elevated GO concentrations.

### Characterizations

The morphological features of rGO@HCFs were observed using a field emission high-resolution electron microscope (ZEISS Sigma 300, Germany). The cross sections and the surface morphologies of the samples were prepared by pulled apart by tweezers. All SEM samples were observed under the 3 kV extra high tension (EHT) after sputter coated with gold particles before observation. Optical absorption performance of the rGO@HCFs were investigated by a UV–vis–NIR spectrometer (Hitachi U4150, Japan) over a range from 200 to 2500 nm. Wettability of rGO@HCFs were performed with a video contact angle meter (JY-82C, Chengde Dingsheng Test Machine Detection Equipment Co., Ltd.). The vaporization enthalpy of bulk water and absorbed water in rGO@HCF_0.06_ were investigated by a Differential Scanning Calorimeter (DSC, HNB-DSC300C, Xiamen Senbei Technology Co., Ltd. China). In addition, the surface temperature behavior of rGO@HCF was monitored by an infrared thermal imaging analyzer (ST9450, Dongguan Wanchuang Electronic Products Co., Ltd., China). A xenon lamp (PLS-SXE300+, China, Beijing Perfectlight Technology Co., Ltd.) simulating indoor sunlight and a radiometer (FZ-A, Beijing Normal University Optoelectronic Instrument Factory) assessed light intensity. The ionic concentration of both seawater and purified water was characterized using an ICPOES (Agilent 5110, USA).

### Evaluation of solar-driven vapor generation performance

A unique apparatus was developed for the indoor evaluation of the solar-driven vapor production capability of rGO@HCFs, in addition to an outdoor assessment rig. Purified water (100 mL) was placed in a glass cup of 4 cm diameter, and a circular PS foam piece, swathed in cotton fabric, was seated within the vessel. With the PS foam positioned roughly 3 cm above the liquid, the cotton extended into the water, ensuring consistent moisture availability for the rGO@HCF. Mass loss induced by evaporation was precisely tracked by a computer-tethered electronic balance (sensitivity of 0.001 g) under a 300W xenon lamp (Beijing Perfectlight Technology Co., Ltd., PLS-SXE300+) equipped with an AM1.5 filter. These experiments were conducted over an hour under stable temperature (23 ± 2 °C) and humidity (25–28%). For outdoor experiment, the indoor setup was housed within an acrylic sealed barrel to guarantee ample sunlight exposure and water collection, performed at Xinzhou Normal University, Shanxi. Solar irradiance was quantified hourly from 8:00 am to 6:00 pm, with a 10-h mean value serving as the representative measure.

### Ethical approval

No animals were killed. There is no ethical approval needed in this work.

## Results and discussion

### rGO@HCF composite membranes

**Figure 1:**
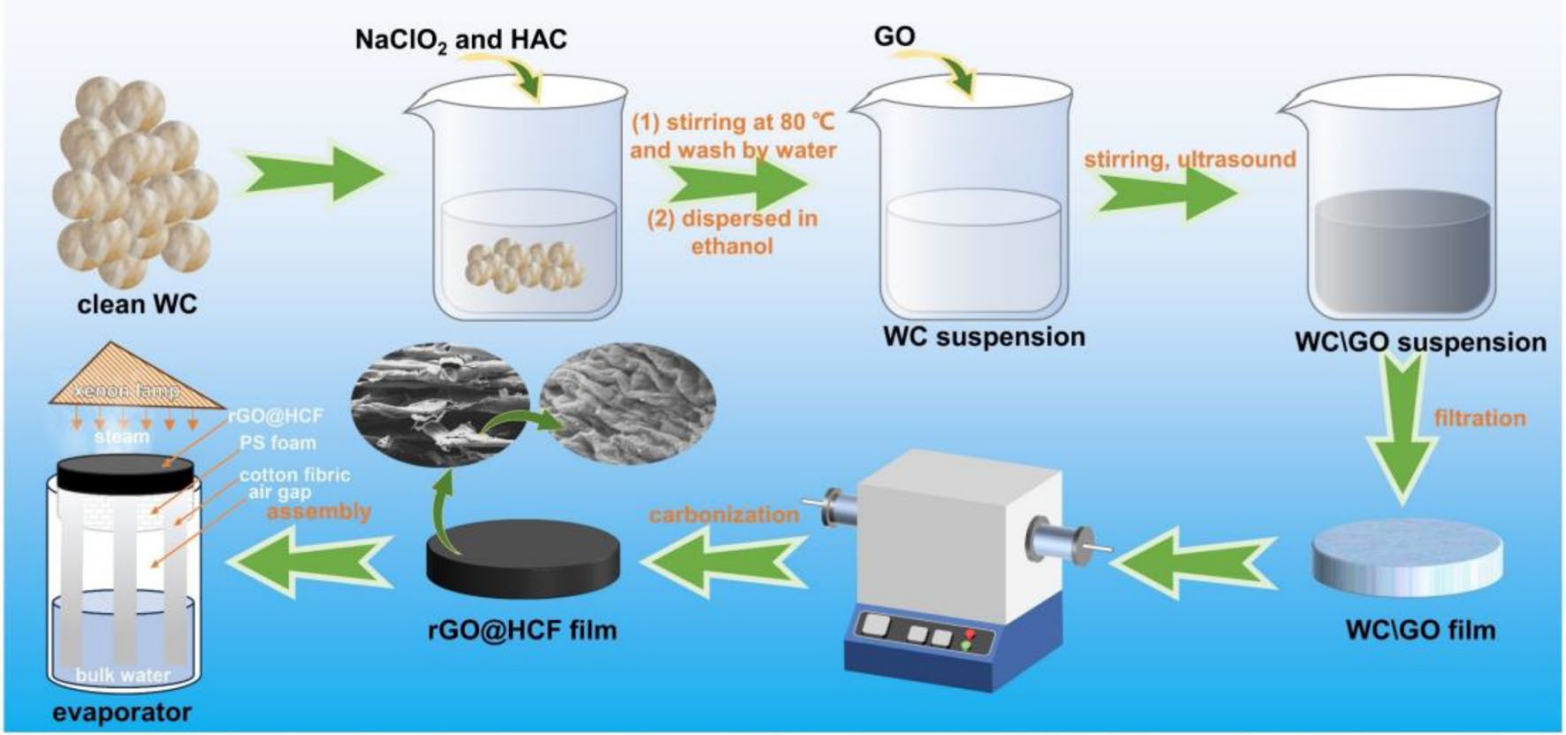
Schematic illustration of preparation process for the WC based carbon films.

**Figure 2:**
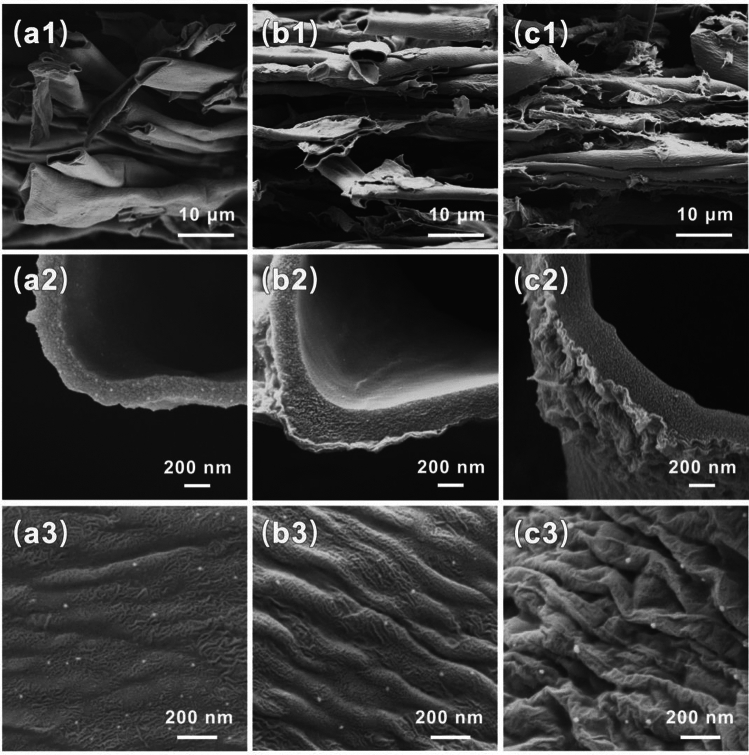
SEM images of rGO@HCF composite membrane, where a, b, c represents HCF, rGO@HCF_0.03_, rGO@HCF_0.06_, 1, 2, 3 represents overall morphology, cross-sectional morphology and surface morphology of rGO@HCF composite membrane, respectively.

As depicted in Fig. 1, rGO@HCF composite films were fabricated employing a vacuum-assisted filtration method. The WC samples were meticulously purified and subsequently subjected to a NaClO_2_ treatment aimed to remove lignin, conducted at 80 °C for a duration of 2 h^14,30^. Post preparation, the WC suspensions were amalgamated with GO solution and thoroughly homogenized within an anhydrous ethanol medium. The resulting mixture was then subjected to vacuum filtration and a carbonization process at an elevated temperature of 900 °C under an inert nitrogen atmosphere for 2 h. According to the references^31,32^, GO would partially transform into reduced graphene oxide (rGO) at high temperatures. Figure 2 presents a comparative analysis of the surface morphology and microstructure between HCF and rGO@HCFs composites. HCF exhibits a typically hollow tubular fiber architecture (Fig. 2a1,a2) adorned with intermittent, low-amplitude, wave-like ridges (Fig. 2a3). These characteristics are consistent with the results of prior investigations^14,33,34^. Utilizing a strategic combination of blending and heat treatment, GO has been successfully enwrapped on the HCF surface, as illustrated in Fig. 2b2,c2. As shown in Fig. 2c3, it is exciting that regular wave-like, undulatory folding microstructures form on the surface of hollow carbon fibers. Notably, the intensity of these microstructures is accentuated with the incremental addition of GO (Fig. 2b3,c3). The genesis of these regular wavy folds likely stems from the adsorptive interaction between GO and WC during the formation of a homogenized suspension, with subsequent GO deployment on the WC hollow fibers surface. High-temperature carbonization induces a differential shrinkage between WC and rGO. Additionally, there is noted that evidence of GO did not completely conform to the surface wrapping of HCF (Fig. 2c1), but instead intermingled with rGO@HCF, serving to bridge and interconnect the rGO@HCF fibers.

**Figure 3:**
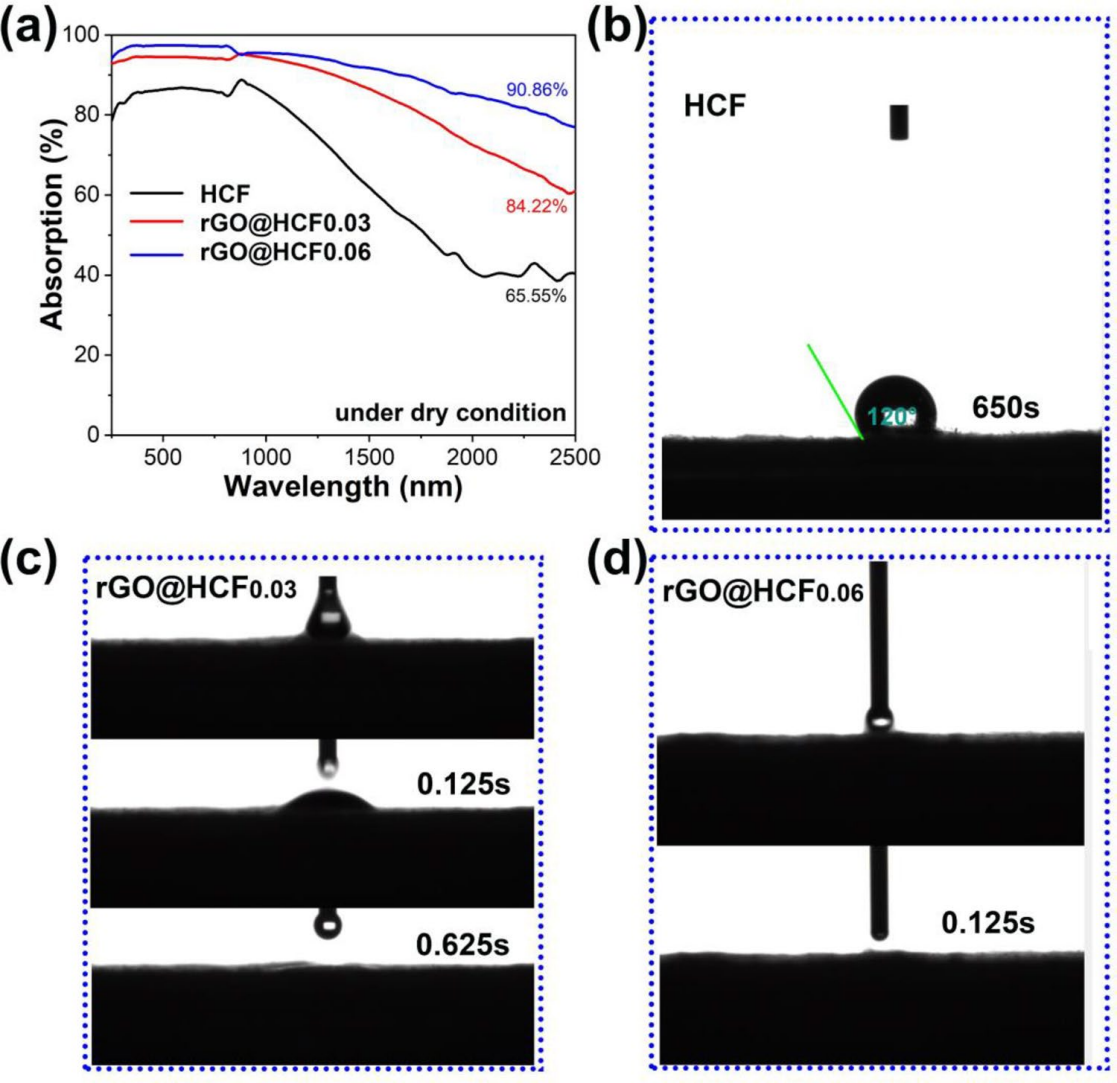
(**a**) UV–vis-NIR absorption spectrum of rGO@HCF composite film; (**b**–**d**) are water drop angle testing pictures of samples HCF, rGO@HCF_0.03_ and rGO@HCF_0.06_, respectively.

As delineated in Table 1, the elemental compositions on the surface of three kinds of evaporator are quantified, revealing that the oxygen content in pristine HCF is registered at 10.92 wt%. This metric notably ascends to 19.36 wt% on the rGO@HCF_0.06_ surface. Wrapped rGO on the HCF has greatly increased the content of oxygen-containing functional groups. The increase of oxygen-containing functional groups will have a beneficial effect on the hydrophilicity of the prepared evaporator, ensuring sufficient supply of interface water during the water vapor production process. The wettability characteristics of the evaporators are captured in Fig. 3b–d. Pristine HCF exhibits pronounced hydrophobicity, which water droplet kept a contact angle of 120° even post 650 s. Conversely, the rGO@HCF composites showcase exceptional super-hydrophilic behavior; for instance, a droplet rapidly disperses across the rGO@HCF_0.03_ sample surface within a mere 0.625 s, while contact instigates instantaneous spreading on the rGO@HCF_0.06_ sample. Such hydrophilic tendencies are in concordance with the increment of oxygen-containing functional groups on the surface of rGO@HCF.

**Table 1:** Surface element content of rGO@HCF composites membrane.

Sample	Elements/weight%		
	C	O	N
HCF	86.88	10.92	2.20
rGO@HCF_0.03_	81.07	17.90	1.03
rGO@HCF_0.06_	80.16	19.36	0.48

The innovatively engineered wave-like and creased micro-to-nanostructures furnish a conducive platform for multifaceted light reflection, thereby amplifying the light absorption efficacy and photothermal transduction proficiency of the evaporator. A comprehensive evaluation, utilizing a UV–vis–NIR spectrophotometer, was conducted to investigate the solar light capture performance of dry condition of the rGO@HCFs. As exhibited in Fig. 3a, light absorption for unmodified HCF is only 65.55% over the extensive UV–vis-NIR spectrum (250–2500 nm). A notable surge is observed when rGO loading varies from 0.03 to 0.06 wt% within the composite, catapulting light absorption increase from 84.22% to a remarkable 90.86%. This corresponds to HCF absorptive gains of 28.5% and 36.6%, respectively. Consequently, the rGO-enriched, wave-like surface topology markedly bolsters the light absorption capabilities of the evaporators, which would enhance the water evaporation rate of the resulting evaporators.

### Photo‑to‑thermal conversion properties of the rGO@HCFs

**Figure 4:**
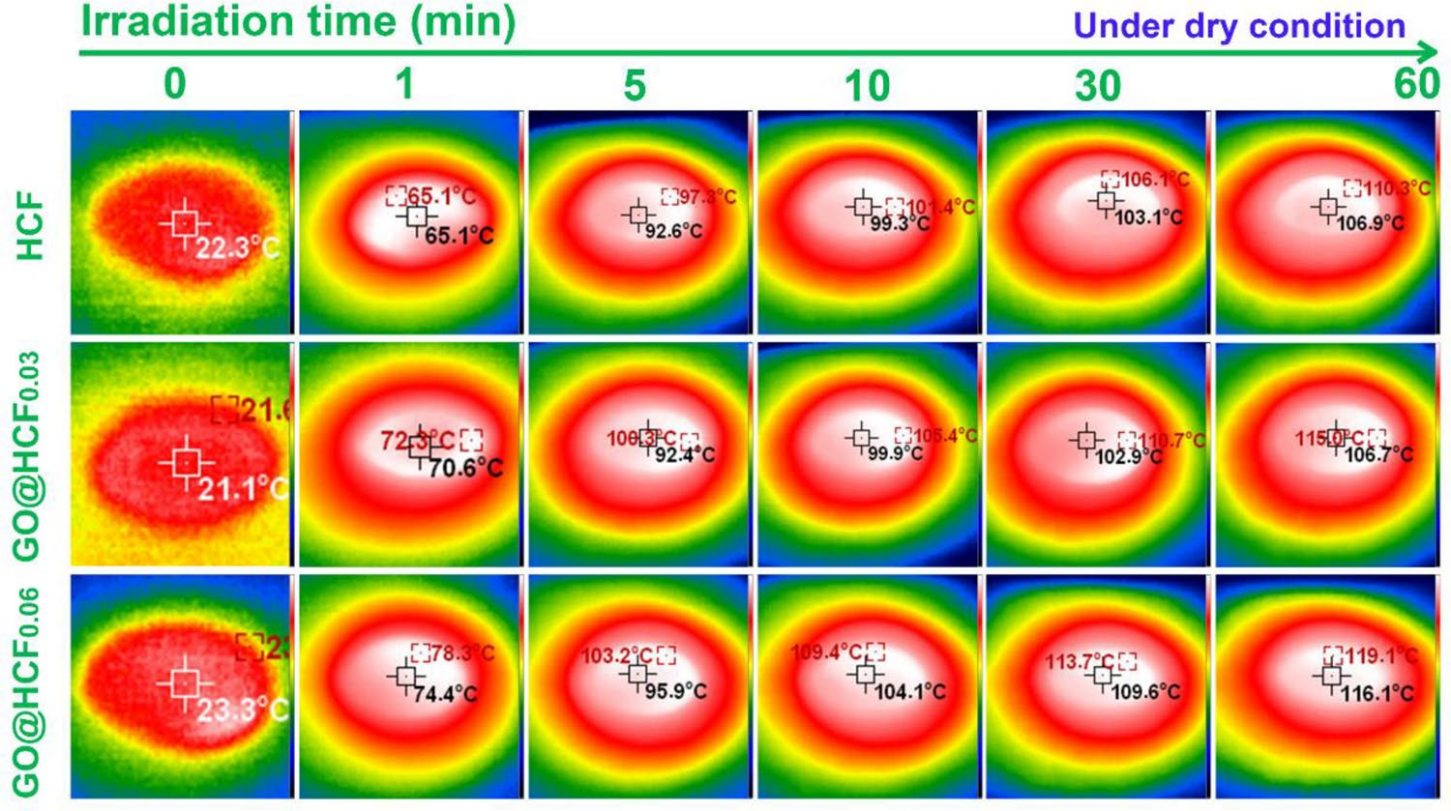
Temperature dependent illumination time for dry surface of HCF, rGO@HCF_0.03_ and rGO@HCF_0.06_ under one sun (1 kW m^−2^) simulated solar illumination intensity.

**Figure 5:**
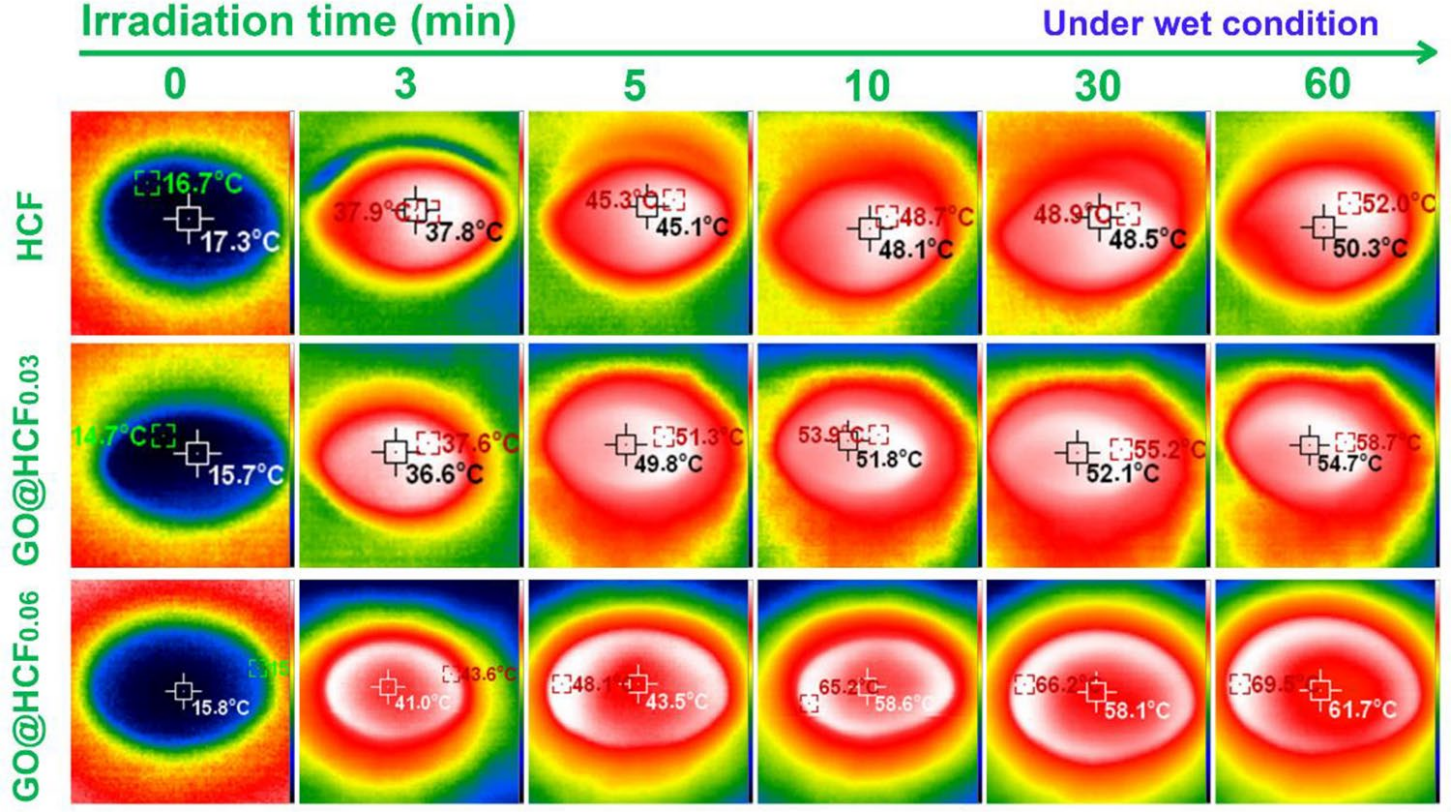
Temperature dependent illumination time for wet surface of HCF, rGO@HCF_0.03_ and rGO@HCF_0.06_ under one sun (1 kW m^−2^) simulated solar illumination intensity.

For comprehensive assessment of the photothermal conversion efficacy of the rGO@HCF evaporators, an infrared thermal imager was employed to record the surface temperature during the one sun irradiation (1 kW m^−2^) for the dry and wet samples. The corresponding results are elucidated in Figs. 4 and 5. As illustrated in Fig. 4, the dry surface temperature of the dry rGO@HCF_0.06_ was swiftly ascended from ambient temperature (23.3 °C) to 78.3 °C within the first minute, with a value higher than that of HCF and rGO@HCF_0.03_ 13.2 °C and 6.0 °C respectively; in the next 4 min, the temperature of the dry rGO@HCF_0.06_ further rise to 103.2 °C, which is also higher than that of HCF and rGO@HCF_0.03_ 5.9 °C and 2.9 °C respectively. Advancing over a span of 55 min, the dry surface temperature of rGO@HCF_0.06_ continued to escalate gradually, stabilizing ultimately at 119.1 °C, which is markedly superior to HCF and rGO@HCF_0.03_ by differentials of 8.8 °C and 4.1 °C, respectively. The above results indicate that with the increase of wavy wrinkled rGO on the surface of HCF, the photothermal conversion performance of the material gradually enhances, and the trend of the photothermal conversion performance is consistent with its light absorption performance (Fig. 3a).

It is necessary to investigate the photothermal conversion performance of the wetted samples owing to its wetted by water when used as an evaporator. The results indicate that the photothermal conversion performance in the wet state is consistent with that in the dry state. For example, under one sun irradiation, the temperature of the wet surface of rGO@HCF_0.06_ quickly rises from room temperature to 43.6 °C within 3 min, which is 5.7 °C and 6.1 °C higher than that of HCF and rGO@HCF0.03, respectively; over the next 7 min, the wet surface temperature of rGO@HCF_0.06_ rapidly increases to 65.2 °C, which is 16.5 °C and 13.9 °C higher than that of HCF and rGO@HCF_0.03_, respectively; in the subsequent 50 min, the wet surface temperature of rGO@HCF_0.06_ slowly rises to 69.5 °C, and the final stable wet surface temperature of rGO@HCF_0.06_ is 17.5 °C and 10.8 °C higher than that of HCF and rGO@HCF0.03, respectively. The above results suggest that with the increase of the wavy-like rGO folds on the HCF surface, the photothermal conversion performance of rGO@HCF gradually enhances during operation, which is consistent with the trend in its light absorption capability (Fig. 3a) and the temperature change in the dry state. Additionally, by comparing Figs. 4 and 5, it is found that when rGO@HCF is at work, its surface temperature is significantly lower than the surface temperature in the dry state, which may be due to the additional heat uptake necessitated by the phase transition of water from liquid to vapor at the rGO@HCF surface during working.

### Efficacy of water vapor generation in rGO@HCF composite membranes

**Figure 6:**
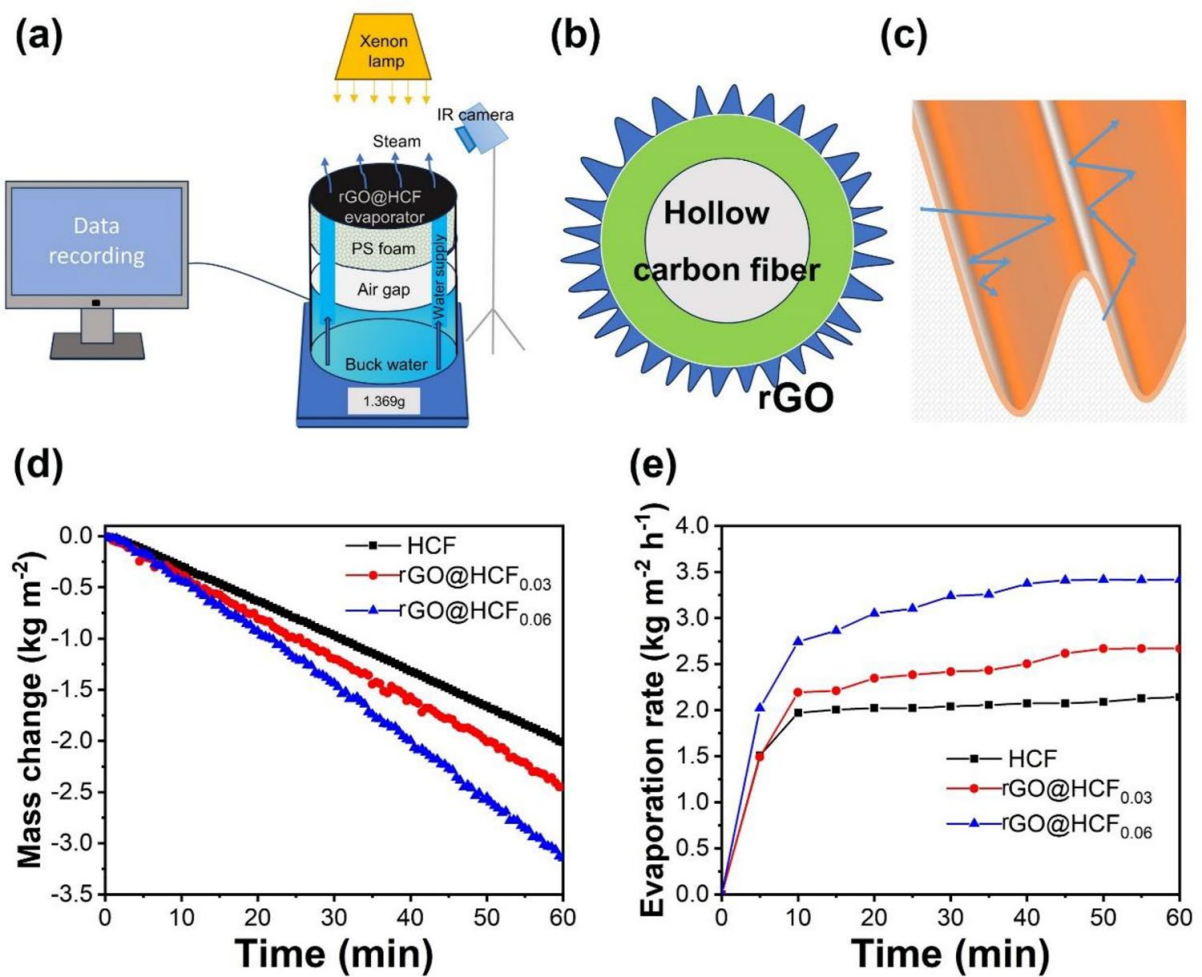
(**a**) Device diagram of indoor solar water steam production test system; (**b**) simulation structure of rGO@HCF; (**c**) reflection way of captured light inside the wavy fold of rGO@HCF surface; (**d**) the dependence of water mass change with the irradiation of time for rGO@HCF-based evaporator under one sun; (**e**) irradiation time dependent evaporation rate of rGO@HCF-based evaporator under one sun.

Assessment of the rGO@HCFs capacity for solar driven water steam generation was evaluated by a home-made experimental apparatus, under ambient conditions (23 ± 2 °C). As presented in Fig. 6a, rGO@HCF samples were placed on a round PS foam wrapped in a cotton cloth, with an air gap of approximately 3 cm between the bottom of the PS and the water surface for avoiding heat loss caused by heating the body water. The cotton cloth was extended into the water to provide sufficient water for evaporation by rGO@HCF; mass changes were recorded by a computer connected -electronic balance, and all indoor tests were completed under one sun irradiation (1 kW m^−2^). The mass change of water in the evaporator is shown in Fig. 6d. Within one hour, the mass reduction per square meter for the evaporator equipped with HCF was 2.01 kg. However, for evaporators equipped with rGO@HCF_0.03_ and rGO@HCF_0.06_, the mass reduction was 2.45 kg and 3.14 kg for per square meter, respectively. Compared to the HCF based evaporator, the vapor production performance increased by 21.9% and 56.2%, respectively. Clearly, the vapor production performance of the rGO@HCF based evaporators is significantly better than that of the HCF based evaporator.

In order to more intuitively evaluate the relationship between the water vapor production rate of the prepared evaporators and the illumination time, the average evaporation rate of the evaporator is calculated at 5-min intervals. The calculated evaporation rate varies with illumination time are shown in Fig. 6e. The evaporation rates of HCF, rGO@HCF_0.03_, and rGO@HCF_0.06_ evaporators quickly reached 1.97 kg m^−2^ h^−1^, 2.19 kg m^−2^ h^−1^, and 2.74 kg m^−2^ h^−1^ respectively within the first 10 min. In the following 50 min; the evaporation rates of HCF, rGO@HCF_0.03_, and rGO@HCF_0.06_ further increased to 2.14 kg m^−2^ h^−1^, 2.67 kg m^−2^ h^−1^, 3.42 kg m^−2^ h^−1^ and reached a stable evaporating rate. Obviously, the evaporation rate of rGO@HCF based evaporators is much greater than that of HCF evaporatorsand the evaporation rate of rGO@HCF evaporators significantly increases with the addition of GO.

**Figure 7:**
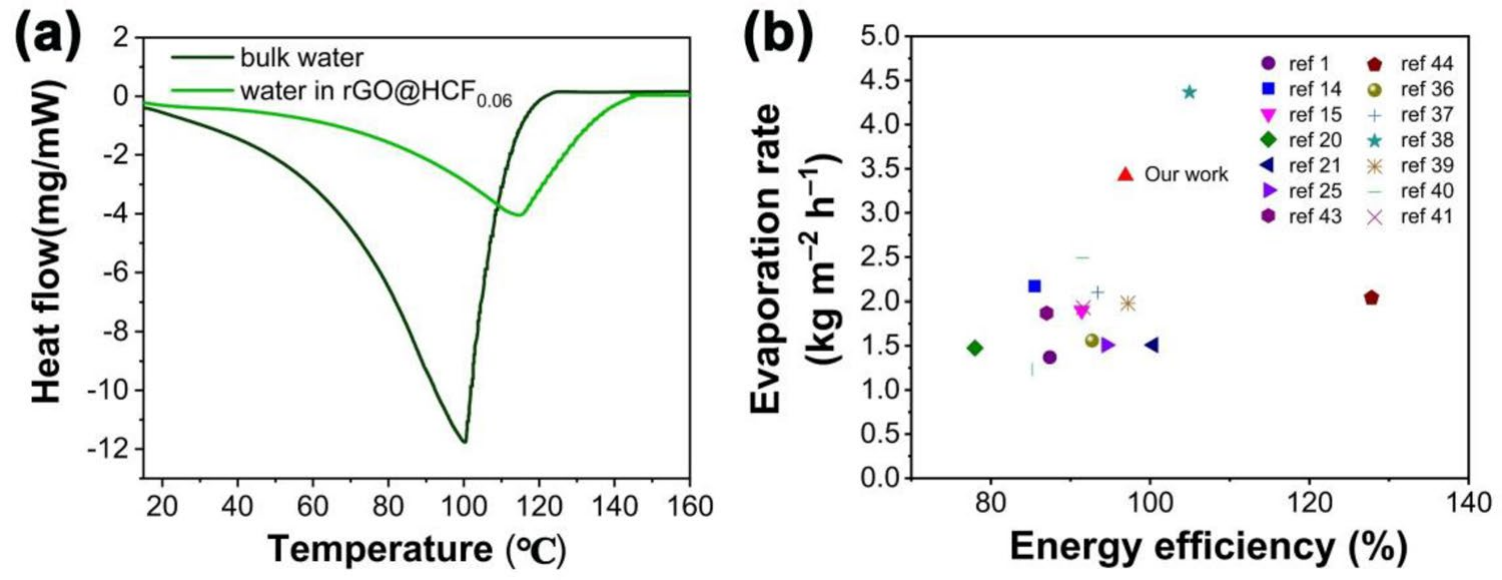
(**a**) DSC curves of the bulk water and absorbed water in rGO@HCF_0.06_; (**b**) Comparison of the evaporation performance of the recent literatures and rGO@HCF_0.06_.

It is worth mentioning that the evaporation rates of rGO@HCF_0.06_ significantly surpass the theoretical limitation value, which is set at 1.592 kg m^−2^ h^−1^ under a light intensity of 1000 W m^−2^ (please refer to the supplementary information for a detailed calculation process). To explore the main reason for high evaporation rates, the vaporization enthalpy of the bulk water and water in rGO@HCF_0.06_ were investigated by means of Differential Scanning Calorimeter (DSC). As shown in the Fig. 7a, the vaporization enthalpy for bulk water was determined to be 2503.92 J g^−1^, which is very close to the reported value^35^. In contrast, the vaporization enthalpy of interfacial water in rGO@HCF_0.06_ was about 1020.54 J g^−1^, which is much lower than that of bulk water. According to the vaporization enthalpy of water in rGO@HCF_0.06_, the corresponding energy efficiency is about 96.9%. Apparently, the exhibited high evaporation rate can be predominantly ascribed to the low evaporation enthalpy associated with adsorbed water in rGO@HCF_0.06_. For better understanding the reasons for the excellent photothermal conversion and energy absorption of rGO@HCF, a simple simulation of the microstructure of the rGO@HCF surface is performed, as shown in Fig. 6b, the captured light will undergo multiple reflections within these wavy folds (Fig. 6c), until it is absorbed and converted into thermal energy, which will heat the interfacial water. Comparing Figs. 5 and 6e, the increasing trend of the evaporation rate for HCF, rGO@HCF_0.03_, and rGO@HCF_0.06_ is consistent with the change in their corresponding wet surface temperatures, indicating that good photothermal conversion performance is the key element for the excellent water vapor production performance of the evaporators. The evaporation rate and energy conversion efficiency have been compared with recently reported materials as shown in Fig. 7b. It is obvious that rGO@HCF_0.06_ exhibits relative high evaporation rate and energy using efficiency^36–42^. The above results show that materials with regular wrinkled structured surface can achieve good light absorption and photothermal conversion performance, leading to the resulting evaporator have good water vapor production performance.

### Outdoor performance and durability

**Figure 8:**
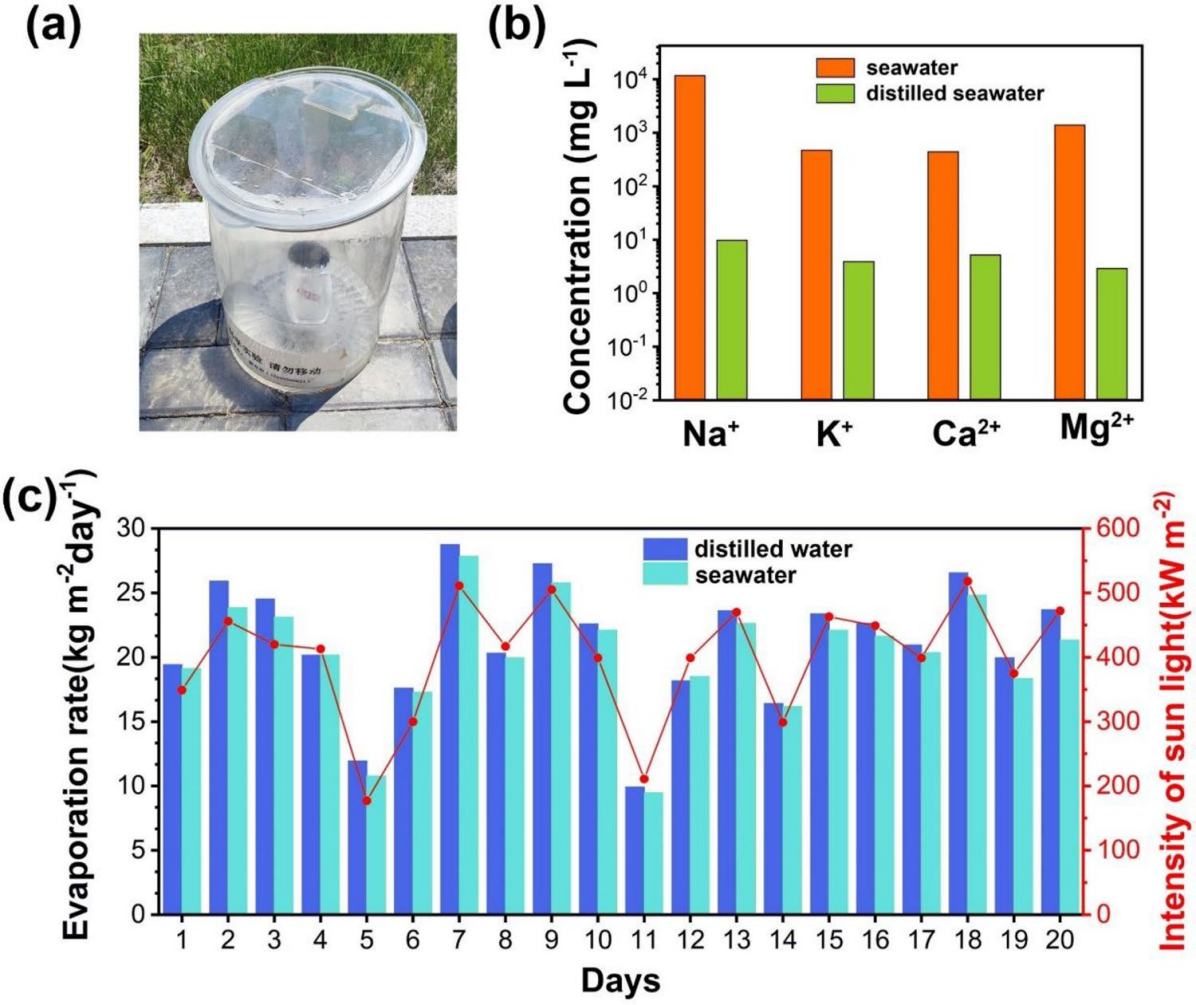
(**a**) The picture of outdoor solar seawater desalination test system; (**b**) The concentration of Na^+^, K^+^, Ca^2+^ and Mg^2+^ irons in the seawater of the Huanghai before and after treatment by rGO@HCF_0.06_ outdoor; (**c**) water steam production performance of rGO@HCF_0.06_ evaporator for 20 days in outdoor.

Working stability and practical performance are important indicators for solar interfacial evaporators. For this reason, an outdoor test setup was designed, as shown in Fig. 8a. The outdoor tests were conducted at the Xinzhou Teachers University in Xinfu District, Xinzhou City, Shanxi Province, and the water evaporation production performance of the rGO@HCF_0.06_ evaporator for deionized water and Yellow Sea seawater was tracked for 20 days, corresponding results shown in Fig. 8b,c. As illustrated in Fig. 8b, after treatment by the rGO@HCF_0.06_, the ion concentrations of Na^+^, K^+^, Ca^2+^, and Mg^2+^ in Yellow Sea seawater dropped from the original 11,779.4 mg L^−1^, 472.3 mg L^−1^, 439.2 mg L^−1^, 1389.9 mg L^−1^ to 9.8 mg L^−1^, 3.9 mg L^−1^, 5.2 mg L^−1^, 2.9 mg L^−1^, respectively. According to the “Standards for Drinking Water Quality” (GB 5749–2006) and the World Health Organization standards, the treated water quality meets the requirements for drinking water^43,44^. As shown in Fig. 8c, the rGO@HCF_0.06_ evaporator performed steadily over a continuous 20 days, with the average daily evaporation amount for deionized water and Yellow Sea seawater reaching 21.2 kg m^−2^ and 20.3 kg m^−2^, respectively, which can meet the drinking water needs of about 10 adults. It should be noted that the daily water production performance of the rGO@HCF_0.06_ evaporator is affected by sunlight intensity, and generally, the water production of the prepared evaporator increases with the enhancement of light intensity, with a fluctuation in the daily evaporation rate of seawater between 9.5 and 27.8 kg m^−2^. According to the above results, the prepared rGO@HCF_0.06_ evaporator exhibits good solar interfacial evaporation performance, service durability and good salt resistance, and these excellent properties are due to the abundance of regularly wavy-like rGO folds on the surface of HCF, which endows rGO@HCF_0.06_ with good photothermal conversion and hydrophilic properties.

## Conclusion

In summary, with the help of combined process of blending and heat treatment, rGO was successfully coated on the surface of HCF and formed a unique wavy-like structure. SEM results show that these wavy-like wrinkles become more pronounced as the amount of GO composite increases, and the surface oxygen functional group content also increases, promoting the hydrophilic and photothermal conversion performances of the corresponding evaporators. DSC findings reveal a significant reduction in the evaporation enthalpy, specifically observing a decline from 2503.92 to 1020.54 J g^−1^ for both bulk water and rGO@HCF_0.06_ adsorbed water. Under one simulated sunlight (1 kW m^−2^) irradiation, the dry and wet surface temperatures of the rGO@HCF_0.06_ evaporator reached 119.1 °C and 61.7 °C, respectively, with a water vapor production rate of 3.42 kg m^−2^ h^−1^; moreover, in outdoor tests, the daily treatment volume of seawater by the rGO@HCF_0.06_ evaporator reached a maximum of 27.8 kg m^−2^ day^−1^, with an average value as high as 20.3 kg m^−2^ day^−1^ over 20 continuous days, and it exhibited excellent durability. The rGO@HCF evaporator features a simple preparation process, low cost, environmental friendliness, and sustainability, which well align with the technical requirements of carbon neutrality. This research may open up a new way for regular wavy-like nano-structure surface preparation and provided a excellent evaporator for solar driven desalination and sewage disposal.

### Supplementary Information

Supplementary Information.

## Data Availability

The data of this manuscript are obtainable from the corresponding author Xidong Suo.
