# Supplementary material for: Hollow carbon fiber wrapped by regular rGO wave-like folds for efficient solar driven interfacial water steam generation
Source: Sci Rep. 2024 Jun 18;14:13997. doi: 10.1038/s41598-024-64144-y (PMC11183090; doi:10.1038/s41598-024-64144-y)
Supplement: Supplementary file 1 — Supplementary Information. [file 41598_2024_64144_MOESM1_ESM.docx]

***Supporting Information***

**Hollow carbon fiber wrapped by regular rGO wave-like folds for efficient solar driven interfacial water steam generation**

Jie Yang^1^, Peiqi Liu^1^, Zhiyuan Fan^2^, Yingying Li^1^, Hongtao Qiao^1^, Xingyu Xu^1^, Sheng Han^3^, Xidong Suo^1, *^

^1^ Department of Chemistry, Xinzhou Normal University, Xinzhou 034000, Shan Xi, China;

^2^ Leicester International Institute, Dalian University of Technology, Panjing 124221, Liaoning, China;

^3^ School of Chemical and Environmental Engineering, Shanghai Institute of Technology, Shanghai, 201418, China

E-mail: xidsuo@126.com (X. Suo)

**Keywords**: solar driven interfacial evaporation; wavy-like fold interface; graphene oxide; hollow carbon fiber; seawater desalination


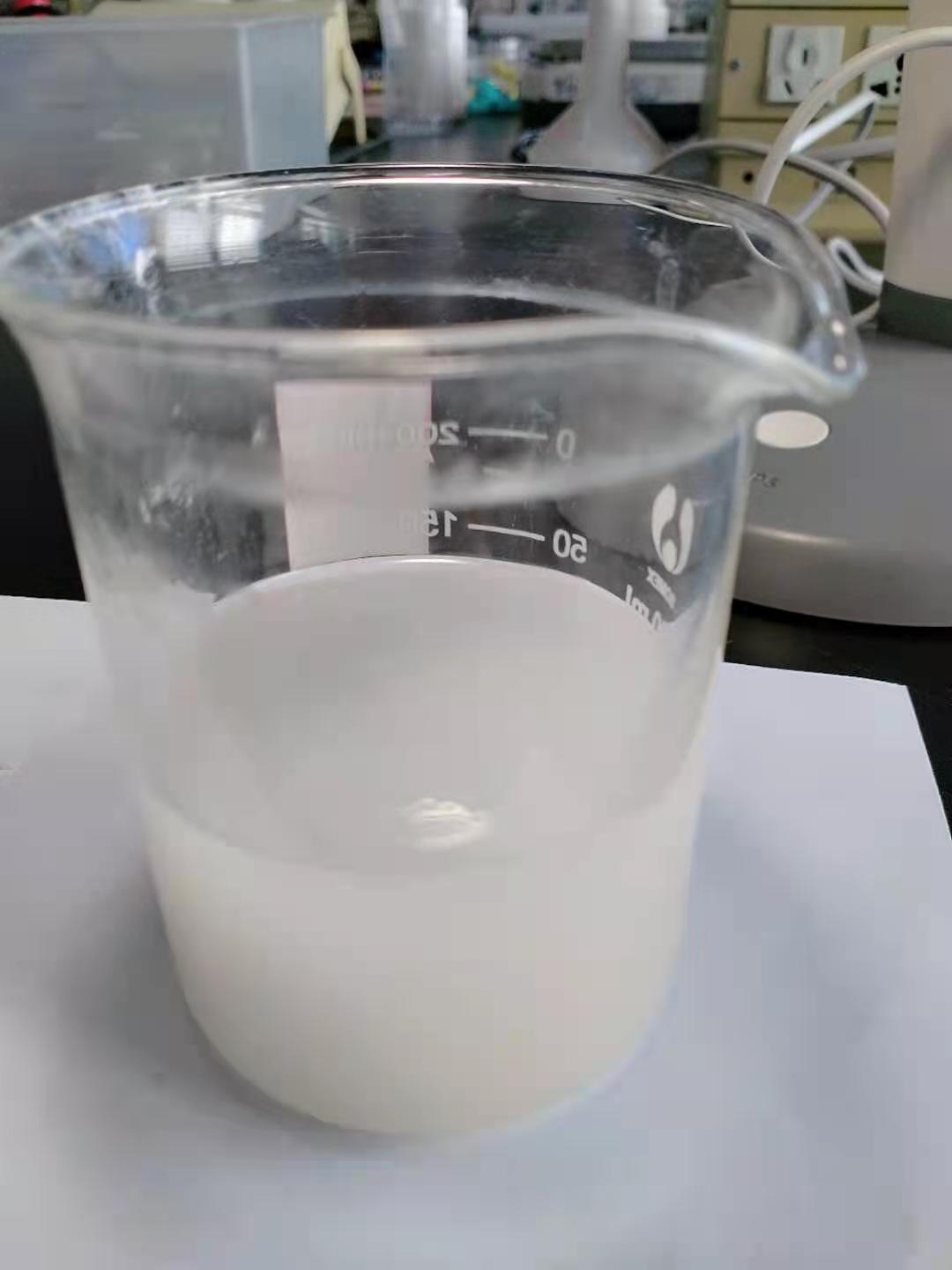


**Figure S1**. The photo of the WC suspension

**Theoretical evaporation velocity calculation**

“The theoretical evaporation speed *v* can be calculated as follows in equation:

$$v=\frac{W\times t}{h_{v}}=\frac{1000\times60\times60}{2257}=1.592kg/m^{-2}$$

Where *W* is light power, which choose to be 1000 W m^-2^, *t* is time (1h), *h_v_* is latent heat of vaporization of water, normally taken to be 2.26 kJ g^-1^ in the region of interest^1^.

1. Gao, M.; Zhu, L.; Peh, C. K.; Ho, G. W., Solar absorber material and system designs for photothermal water vaporization towards clean water and energy production. *Energy & Environmental Science* **2019,** *12* (3), 841-864.
